# Unveiling soil microbial diversity through ultra‐deep short‐read metagenomic sequencing and co‐assembly

**DOI:** 10.1002/imt2.70075

**Published:** 2025-08-18

**Authors:** Pi Lærke Johansen, Ioanna Chatzigiannidou, Lelde Berzina, Karsten Kristiansen, Susanne Brix

**Affiliations:** ^1^ Department of Biotechnology and Biomedicine Technical University of Denmark Lyngby Denmark; ^2^ Department of Biology University of Copenhagen Copenhagen Denmark

## Abstract

By combining ultra‐deep short‐read shotgun metagenomic sequencing with 5‐sample co‐assembly across 600 agricultural soil samples, we significantly enhanced the representation and recovery of microbial communities in both clay and sandy soils. Despite an average of 107 Gb clean reads per sample, projections indicated that 1–4 Tb per sample would be required to capture 95% of the microbial community. Co‐assembly of five biological replicates markedly improved metagenomic recovery, yielding up to 3.7× more metagenome‐assembled genomes, up to 95% more unique genes, and broader recovery of prokaryotic phyla compared to single‐sample assemblies.

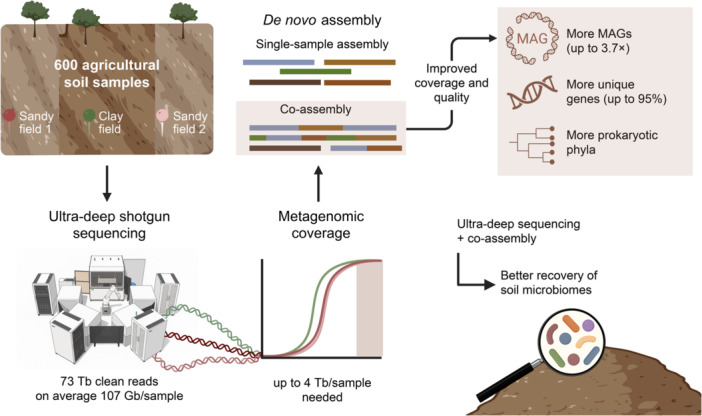

To the Editor,

Soil harbors a rich microbiota that hugely impacts the soil ecosystem and plays important roles in biogeochemical cycles. One gram of soil contains approximately 10^10^ microorganisms, making it the most complex and diverse natural microbial environment on Earth [1, 2]. The soil microbiota is a cornerstone in One Health [3], and understanding its complexity is fundamental in combating biodiversity loss and climate change [4]. In addition, the vast microbial diversity found in soil represents a major genetic resource, with potential industrial and pharmaceutical applications [5]. This resource remains largely untapped, given that only a very small fraction of soil microorganisms has thus far been cultured [6].

Culture‐independent techniques, particularly shotgun metagenomics, enable the study of the “uncultivable” fraction of complex communities. Still, commonly applied metagenomic sequencing depths are far from enough to capture the huge diversity of soil microbes [7]. Thus, metagenomic studies of soil generally suffer from poor classification of the communities [8, 9]. Computational analysis of metagenomic data involves either reference‐based or *de‐novo* assembly‐based approaches. Reference‐based approaches result in poor classification of soil metagenomes due to a limited representation in the databases exemplified by MetaPhlAn4, which enables classification of less than 10% of metagenomic reads from soil [10, 11]. *De‐novo* assembly‐based approaches are database‐independent and essential for the discovery of new microbial species, but are challenged by the extreme diversity of soil, resulting in low metagenomic coverage and limited sequence assembly, hampering the generation of metagenome‐assembled genomes (MAGs) [7].

Based on the assumption that deeper sequencing enhances recovery of low‐abundance taxa and rare functional genes across agricultural soil types, we here examined the influence of sequencing depth on the detection and functional characterization of microbial genes and genomes based on 600 clay and sandy soil samples. In parallel, we evaluated assembly strategies to optimize gene and genome reconstruction for comprehensive soil metagenome analyses.

### The extreme complexity of soil microbial communities

We compared metagenomic coverage across various environments using publicly available metagenomic sequencing data (Table S1). Sequencing depth varied from 1.5 to 42 gigabases (Gb) and was on average four times greater for soil samples than for non‐soil samples (Figure S1A). Only human gut samples reached near‐complete coverage (NCC > 95%, Figure S1B), while soil sample coverage reached merely a coverage of 34%–41% despite much higher sequencing depths (Figure 1A). This low coverage is due to the extreme genomic diversity in soil (Figure 1B), making it difficult to obtain a good representation of the entire community. Projections of Nonpareil curves indicated that it would require 0.9–4.6 terabases (Tb) of clean forward reads per sample to achieve NCC for soil, which is more than 1500‐fold deeper sequencing than needed for human gut samples (Figure 1C). Such extremely high sequencing depths are hard to achieve with current technologies, and even if possible, would make the cost of multi‐sample studies and computational requirements prohibitive.

**FIGURE 1:**
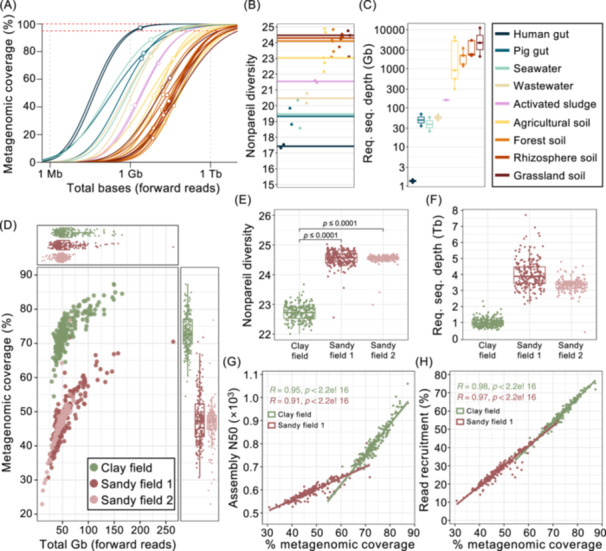
Soil constitutes a very diverse microbial environment, with low metagenomic coverage greatly impacting assembly quality. (A) Nonpareil curves, showing the estimated metagenomic coverage as a function of the sequencing depth. Points represent the actual sequencing depth and estimated coverage of each sample, while lines represent projected and rarefied sequencing depths. (B) Nonpareil diversity of each sample. Lines represent the median diversity of the samples from each environment. (C) Required sequencing depth to achieve near‐complete coverage (NCC > 95%) for each sample. (D) Metagenomic coverage and total size of forward reads in gigabases (Gb) for each sample. (E) Nonpareil diversity, and (F) estimated required sequencing depth to reach near‐complete coverage (NCC > 95%) for each of the samples in the three fields. *De‐novo* assembly was carried out for each of the samples in clay field and sandy field 1, and the resulting (G) assembly N50 and (H) percentage of reads mapping to the >2000 bp assembly contigs are correlated to the metagenomic coverage. (C, E, F) Horizontal lines indicate the median; box boundaries indicate the interquartile range; whiskers represent values within 1.5× the interquartile range of the first and third quartiles. (G, H) Lines show a linear regression, *R* and *p* values are based on Pearson correlations.

### High microbial diversity of agricultural soil leads to low assembly quality due to low metagenomic coverage even at ultra‐deep sequencing depth

To compare and acquire important information on the highly diverse microbial communities of agricultural fields using manageable ultra‐deep short‐read sequencing, we collected 600 soil samples from both clay and sandy soil types. Samples were sequenced to achieve, on average, 107 Gb clean reads per sample, ranging from 23.98 to 588.39 Gb per sample. Despite overall similar sequencing depths, the agricultural fields exhibited huge differences in metagenomic coverage (Figure 1D). The clay field was less diverse (Figure 1E), and reached, on average, 73% coverage with 51.2 Gb clean forward sequencing reads, whereas the microbial more diverse sandy fields reached a metagenomic coverage of 47% with 45.1 and 47.5 Gb of clean forward sequencing reads, respectively. Projection of Nonpareil curves (Figure S1C) indicated that reaching an NCC would require, on average, 1.0 Tb of clean forward reads from the clay field, and 3.9 and 3.4 Tb on average for the two sandy fields, respectively (Figure 1F).

We sought to evaluate the effect of metagenomic coverage on the process of contig assembly, where we analyzed samples from clay field and sandy field 1 as sandy field 2 had similar coverage values to sandy field 1. Assemblies revealed a strong positive correlation between metagenomic coverage and assembly N50, implying that from samples with higher coverage, longer contigs were generated (Figure 1G). When mapping the reads to the assembly contigs >2000 bp in length, we obtained the read recruitment, i.e., the proportion of assembled reads used for downstream analysis. We observed that, similarly to the N50, read recruitment increased with the metagenomic coverage (Figure 1H). Accordingly, due to the higher metagenomic coverage in the clay field, we observed a higher assembly N50, more >2000 bp contigs, and higher read recruitment as compared to the sandy field (Figure S1D–F). However, the read recruitment was quite low for both fields, with 57% from the clay field and 27% from sandy field 1. This means that even for the less diverse clay field, almost half of the sample was lost during the contig assembly step due to low read coverage and consequently inadequate overlap in the reads. Accordingly, despite the deep sequencing effort, a large portion of metagenomic data was unavailable for gene prediction and reconstruction of microbial genomes. To address this, we compared single versus co‐assembly approaches, demonstrating the superiority of co‐assembly for achieving optimal assembly of reads for downstream analysis of highly complex soil samples.

### Co‐assembly of soil samples improves metagenomic coverage, assembly quality, and recovery of microbial genomes and genes

To evaluate the effect of single versus co‐assembly, we performed *in silico* combinations of 2–8 biological replicates, ranging from 61 Gb to 569 Gb of combined clean forward metagenomic reads, and estimated the metagenomic coverage of each combined sample. This revealed that co‐assembly improved the metagenomic coverage considerably, where combining samples increased coverage from on average 47% to 72% for the sandy field and up to 89% for the clay field when seven samples were combined (Figure 2A). The curve when combining samples showed a saturation, similarly to the curve when considering combined sequencing depth (Figure S2A), which was approached after combining 4–5 samples, where additional added samples had minimal impact on coverage relative to the added complexity. We therefore compared co‐assembly using 1–5 samples per soil type to evaluate the impact on assembly quality. Read recruitment improved gradually when including more samples in the assembly (Figure 2B), indicating that increased coverage directly improved read recruitment, and thus a markedly better assembly quality for co‐assemblies (Figure S2B). We next compared single versus 5‐sample co‐assemblies in a total of 340 samples, with average combined sequencing depths of 302 Gb (clay) and 273 Gb (sandy). Compared to single‐sample assemblies, co‐assembly improved N50 from 784 to 1187 (51%) for the clay field and from 589 to 778 (32%) for the sandy field (Figure S2C), signifying that co‐assemblies produced longer contigs that can be used downstream for recovery of microbial genes and genomes. In addition, co‐assembly increased the read recruitment from 57% to 77% (35% improvement) for the clay field and from 27% to 52% (91% improvement) for the sandy field (Figure 2C).

**FIGURE 2:**
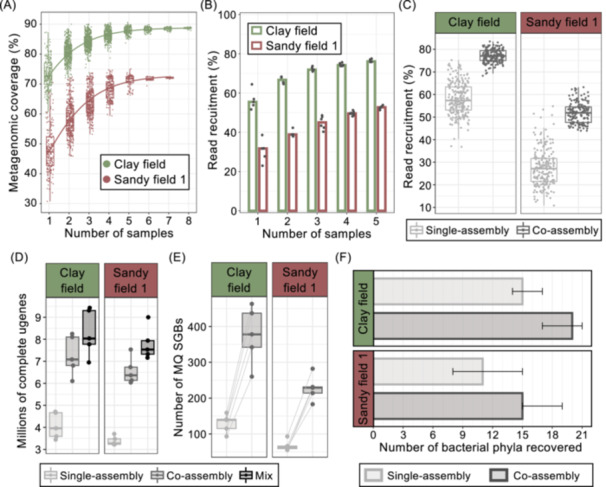
Co‐assembly increases metagenomic coverage and assembly quality and improves recovery of microbial genomes and genes from soil samples. (A) The relationship between the number of samples added in a co‐assembly and the resulting metagenomic coverage. Logistic regressions are fitted to the medians of the coverage values for each of the number of samples per field. (B) Assemblies were carried out for five samples per field. Samples were single‐assembled or assembled in 2, 3, 4, and 5‐sample co‐assemblies. The percentage of reads mapping to >2000 bp assembly contigs increased with the number of samples used in the assembly. Bars show the median percentage of reads mapped. 5‐sample co‐assemblies was carried out for 340 samples and was compared with single assemblies based on the (C) percentage of reads mapped to >2000 bp assembly contigs, and the number of (D) complete unique genes in millions, (E) medium quality (MQ) species‐level genome bins (SGBs), and (F) phyla recovered from single‐ and co‐assemblies, respectively. (A, C–E) Horizontal lines indicate the median; box boundaries indicate the interquartile range; whiskers represent values within 1.5× the interquartile range of the first and third quartiles. (E) Lines connect points representing the same group of samples single‐assembled and co‐assembled. (F) Bars represent the median number of phyla recovered, while whiskers represent the minimum and the maximum values.

To understand the impact of improved assembly quality from co‐assemblies on the recovery of microbial genes and genomes, we recovered MAGs and predicted genes from 5× 5‐sample co‐assemblies per soil type, and compared them to single‐sample assemblies of the same samples. Co‐assembly improved both the recovery of total (Figure S3A) and complete unique genes, and resulted in a 79% (3.96M to 7.09M, clay field) and a 95% (3.27M to 6.37M, sandy field) increase in the number of recovered complete unique genes, which can be further improved by combining the two assembly approaches (Figure 2D). Co‐assembly also increased the number of medium‐quality (MQ, >50% completeness and <10% contamination) and near‐complete (NC, >90% completeness and <5% contamination) species‐level MAGs recovered from both fields. Co‐assembly recovered 2.7 times more MQ MAGs from the clay field and 3.7 times more from the sandy field than single assemblies (Figure 2E). NC MAG recovery was also improved from 33 to 60 for the clay field and from 10 to 30 for the sandy field (Figure S3B). At the strain level, co‐assembly recovered more MQ MAGs than single assemblies (Figure S3C). However, for the clay field, co‐assemblies recovered fewer NC strain‐level MAGs in three out of four cases, while this number was substantially increased for the sandy field (Figure S3D), suggesting the loss of strain‐level resolution when there are multiple strains within the same species. Co‐assembly resulted in the recovery of more prokaryotic phyla (Figure 2F) and genera (Figure S3E) compared to single assemblies. Some prokaryotic phyla, such as Micrarchaeota, SAR324, Armatimonadota, and Verrucomicrobiota, were consistently recovered from co‐assemblies but were never or only rarely recovered from any single assembly (Figure S3F).

We corroborate earlier evidence that soil harbors far greater microbial diversity than other microbial environments [7]. Increasing the sequencing depth up to 588 Gb enhanced metagenomic coverage, yet captured only 47%–73% of the microbial community. This coverage, even at ultra‐deep sequencing, underscores the complexity of soil microbiomes and the limitations of short‐read metagenomics in fully resolving the microbial diversity in agricultural soil, which helps explain the incomplete characterization reported in previous studies [8, 9].

Low coverage poses significant challenges for *de‐novo* assembly, as sparse genome representation leads to poor read overlap and fragmented assemblies. Consistent with this, we observed a strong positive correlation between metagenomic coverage and assembly quality. Yet, even in the less diverse clay field, only 57% of the reads could be assembled using single‐sample assemblies, resulting in substantial data loss. This suggests that in highly diverse soils, deeper sequencing may not proportionally enhance resolution, emphasizing the diminishing returns of sequencing depth alone in resolving highly diverse microbiomes, and pointing to the value of complementary approaches such as co‐assembly to maximize data utility.

The co‐assembly versus single‐sample assembly approach significantly increased gene and MAG recovery and enabled the detection of a broader range of prokaryotic phyla, including rare or previously undetected lineages. The improvement in phyla recovery with co‐assembly was also earlier observed in tropical soils [12]. As co‐assembly nearly doubled the number of recovered complete genes—potentially encoding enzymes or metabolites of industrial or ecological relevance—and yielded up to 3.7 times more MAGs, it clearly enhances our capacity to link taxonomy with genome functionality. However, single assemblies recovered more near‐complete MAGs at the strain‐level in some cases, reflecting a trade‐off between overall MAG yield and strain resolution when multiple strains co‐occur. This merging effect has been noted in other studies [13, 14]. However, contigs from single samples may also be chimeric [15], and co‐assembly does not necessarily increase overall chimerism [16], so the choice between single and co‐assembly will largely depend on study goals. Moreover, computational resources may also be a factor, as co‐assembly required, on average, twice the processing time. Tools like StrainPhlan [17] can supplement either approach to improve strain resolution, although read‐based methods currently remain limited in soil due to low classification rates.

Assembly quality may be further improved by incorporating long‐read sequencing to reduce fragmentation, although this does not always improve MAG recovery [18, 19]. Hybrid assemblies, combining long‐ and short‐read sequencing data, show promise in overcoming assembly limitations in complex, low‐coverage environments [18, 20], but the high cost and low throughput of long‐read sequencing currently limit their use in large‐scale studies. As long‐read technologies advance, they may become more accessible, enabling broader application in soil metagenomics.

Our study provides valuable insights into the influence of sequencing depth and assembly strategies in soil metagenomics. By leveraging ultra‐deep short‐read metagenomic sequencing and 5‐sample co‐assembly, we achieved up to 52% higher metagenomic coverage, translating to a 91% increase in assembled reads, and thus the recovery of more comprehensive microbial gene and genome datasets, enhancing our understanding of soil microbial diversity and its ecological functions.

Procedures for sample collection, DNA extraction, sequencing, data processing, and analyses are available in the Supplementary Information.

## AUTHOR CONTRIBUTIONS

**Pi Lærke Johansen**: Conceptualization; methodology; data curation; formal analysis; visualization; writing—original draft; writing—review and editing. **Ioanna Chatzigiannidou**: Conceptualization; methodology; data curation; supervision; writing—original draft; writing—review and editing. **Lelde Berzina**: Methodology; writing—review and editing. **Karsten Kristiansen**: Conceptualization; methodology; investigation; funding acquisition; project administration; writing—review and editing; resources. **Susanne Brix**: Conceptualization; methodology; investigation; writing—original draft; writing—review and editing; project administration; resources; supervision.

## CONFLICT OF INTEREST STATEMENT

The authors declare no conflicts of interest.

## ETHICS STATEMENT

No animals or humans were involved in this study.

## Supporting information


**Figure S1:** Metagenomic sequencing depth and coverage vary in different environments and greatly impact assembly quality.
**Figure S2:** Co‐assembly increases metagenomic coverage and read recruitment to assembly.
**Figure S3:** Co‐assembly improves recovery of microbial genomes and genes.

**Table S1:** Public metagenomic samples included in Figure 1A–C.
**Table S2:** Sequencing samples and their grouping for *in silico* combination and co‐assemblies.

## Data Availability

Raw sequencing data have been deposited in the European Nucleotide Archive under accession number PRJEB83729 (https://www.ebi.ac.uk/ena/browser/view/PRJEB83729). The data and scripts to create all figures are saved in GitHub https://github.com/piljoha/SoilUltraDeep. Supplementary materials (methods, figures, tables, and graphical abstract) may be found in the online DOI or iMeta Science http://www.imeta.science/.
