# Supplementary material for: Unveiling soil microbial diversity through ultra‐deep short‐read metagenomic sequencing and co‐assembly
Source: Imeta. 2025 Aug 18;4(5):e70075. doi: 10.1002/imt2.70075 (PMC12527996; doi:10.1002/imt2.70075)
Supplement: Supplementary file 1 — Figure S1: Metagenomic sequencing depth and coverage vary in different environments and greatly impact assembly quality. Figure S2: Co‐assembly increases metagenomic coverage and read recruitment to assembly. Figure S3: Co‐assembly improves recovery of microbial genomes and genes. [file IMT2-4-e70075-s001.pdf]

Supporting Information to

Unveiling soil microbial diversity through ultra-deep short-read metagenomic sequencing and co-assembly

Running title: Combined ultra-deep metagenomics and co-assembly reveal soil microbial diversity

Pi Lærke Johansen[1], Ioanna Chatzigiannidou[1], Lelde Berzina[2], Karsten Kristiansen[2*], Susanne Brix[1*]

[1]Department of Biotechnology and Biomedicine, Technical University of Denmark, Kgs. Lyngby 2800, Denmark

[2]Department of Biology, University of Copenhagen, Copenhagen 2200, Denmark

[*]Correspondence: kk@bio.ku.dk (Karsten Kristiansen), sbrix@dtu.dk (Susanne Brix)

METHODS

Sample collection

Soil samples were collected from three different agricultural fields in Denmark: Clay field (GPS coordinates in decimal degrees: 57.195949, 9.792213), Sandy field 1 (GPS: 57.149500, 10.233722), and Sandy field 2 (GPS: 55.781778, 11.524833). Samples were collected with a minimum of 5 meters between each sampling site. 25 grams of soil were sieved to a maximum diameter of 2 mm to eliminate larger particles. The sample was then transferred to a 15 mL tube together with PBS and 20% glycerol solution and stored at –80 °C until further use.

DNA extraction

Before extraction, samples collected in PBS and 20% glycerol solution were spun for 10 min at 4000 × g, room temperature. After the removal of supernatant, 250 mg of soil sample was transferred to MN Bead Tube Type A, and DNA extractions were conducted using the NucleoSpin Soil, Mini kit (740780.250, Macherey-Nagel GmbH&Co, Germany). The sample material was resuspended in SL1

lysis buffer, supplemented with the Enhancer XS, followed by mechanical disruption on a Precellys® Tissue homogenizer (Bertin Technologies SAS, France). PCR inhibitors and proteins were precipitated with SL3 lysis buffer and pelleted by 10 min centrifugation at 11,000 × g. Next, samples were loaded on NucleoSpin® Soil columns and subsequently washed with Binding buffer SB and Wash Buffers SW1/SW2 to remove residual humic substances and inhibitors. After 10 min of silica membrane drying, samples were eluted in 80 °C pre-warmed SE Elution buffer (5 mM Tris/HCl, pH 8.5) and incubated for 10 min at room temperature, finished with 2 min centrifugation at 11,000 × g. Samples were stored at –80 °C until further analysis.

Shotgun metagenomic sequencing

Libraries for metagenomic short-read shotgun sequencing were constructed using MGIEasy FS DNA Library Prep set (1000017572, MGI Tech Co., Ltd., China). In brief, library preparation included DNA shearing for 8 min at 30 °C and magnetic bead-based size selection, end repair, and adapter ligation followed by magnetic bead cleanup, PCR, and magnetic bead-based size selection of the final product. Final library concentration and fragment size were determined by Qubit 1X dsDNA HS Assay Kit (Q33231, ThermoFisher, USA) and High Sensitivity D1000 Assay Kit (Agilent Technologies, 5067-5584, USA), respectively. Pooled, circularized, and barcoded libraries were used for DNA nanoball (DNB) formation and further analysed on DNBSEQ-T10×4RS (MGI Tech) using DNBSEQ-T10×4RS High-throughput Sequencing Set (FCL PE150) (940-000100-00, MGI Tech) according to manufacturer's instructions.

Read processing

Removal of adapters and quality trimming were performed with trim_galore [1] v0.6.4, using cutadapt [2] v3.5 (clay field and sandy field 1) or v4.4 (sandy field 2), with a stringency of 5 bp for the overlap with adapter sequence required to trim a sequence, removing 15 bp from the 5' end of all reads, and keeping reads with a minimum length of 50 bp after trimming. After the quality control, we obtained clean metagenomic reads with a total size of 73.36 terabases (Tb), and with an average of 107.26

gigabases (Gb) data per sample (ranging from 23.98 to 588.39 Gb).

Metagenomic coverage estimation

Metagenomic coverage was determined from filtered forward reads with Nonpareil [3] v3.4.1, using the *k*-mer algorithm with default options except that the maximum number of reads to use as query was increased to 100,000 to improve the accuracy of coverage estimations. The metagenomic coverage, Nonpareil sample diversity, and the required sequencing depth to achieve near-complete coverage (NCC), was determined with the Nonpareil R-package v3.5.3. Nonpareil saturation curves were plotted with the Nonpareil R-package.

In silico combination of samples

For the clay field and sandy field 1, each sample was grouped together with samples with the same biological conditions, *i.e.*, biological replicates from different physical locations on the same field (Table S2). Each of the groups contained 3–9 samples. For each of the groups, the forward read fastq files were combined with all possible combinations of forward read fastq files of the samples in the same group. For these combined fastq files, nonpareil [3] v3.4.1 was run as described above. For 24 combinations of 6–9 samples, nonpareil failed to run, likely due to the size and complexity of the input, and these combinations were therefore excluded from further analysis. Metagenomic coverage of each *in silico* combination of samples was estimated with the nonpareil R package v3.5.3.

De novo assembly

We carried out assemblies on the HPC system Computerome (Danish National Life Science Supercomputing Center), using nodes with 2 CPUs, with 20 cores/CPU, and 1536 GB of memory.

Filtered reads were assembled with megahit [4] v1.2.9. Single-sample assemblies were carried out with default settings, using the --presets meta-large, as recommended for complex metagenomes such as soil. The peak memory usage of single assemblies with these settings were up to 927 GB RAM, with a median of 244 GB RAM, and the time usage was up to 143 h (6 days) with a median of 61 h. Co-

assemblies were always based on the combination of samples from biological replicates, as suggested previously [5]. For one group of each condition, co-assemblies of two random combinations of 2, 3, and 4 samples were carried out. 5-sample co-assembly was carried out for all groups containing 5 samples. Co-assembly was performed using a limited *k*-mer range (27,47,67,87,107,127,137) to decrease processing time and memory consumption. These co-assemblies required up to 1.5 TB RAM, and the time usage was up to 244 h (10 days) with a median of 116 h (5 days). For downstream use, the contigs were filtered to keep only those longer than 2000 bp. The read recruitment to assemblies was determined by mapping filtered reads to the final contigs with coverM [6] v0.6.1 using the contig mode.

Prediction of genes and recovery of metagenome assembled genomes

Five groups from each field were chosen for downstream recovery of microbial genes and genomes. For each group, genes and MAGs were recovered for both the 5-sample co-assembly of all five samples in the group, and for each of the five single assemblies of each sample in the group. ORFs were predicted with prodigal [7] v2.6.3 using the meta option for metagenomes. The predicted ORFs were clustered into unique genes based on > 95% nucleotide similarity and were dereplicated with the linclust function of mmseqs2 [8] v15.6f452 using default options, but increasing the minimum fraction of aligned residues to 0.9 and the number of *k*-mers per sequence to 80 to increase sensitivity. To recover microbial genomes from each assembly, the filtered reads from each sample were mapped to the contigs of the single assembly, and to the co-assembly of the group of samples with bowtie2 [9] v2.5.3 to create SAM files, which were converted to BAM format, mapped, and sorted with samtools [10] v1.18. MAGs were produced with VAMB [11] v4.1.3 with the vae model for each assembly, providing the assembly fasta and the BAMs for the sample(s) included in the assembly. Bins with a minimum size of 200,000 bp were kept. Bin quality was evaluated with checkm2 [12] v1.0.2 using the general model, and MAGs from the co-assembly of each group, and all of the single-assemblies of each group respectively were dereplicated to 95% percent Average Nucleotide Identity (ANI) similarity (species-level MAGs) and 99.9% ANI similarity (strain-level MAGs) with dRep [13] v3.4.5 using fastANI [14] v1.33 for secondary cluster comparisons, keeping bins with a minimum completeness of 50% and maximum

contamination of 10%. MAGs with > 50% completeness and < 10% contamination was denoted as medium-quality (MQ) MAGs, and MAGs with > 90% completeness and < 5% contamination was denoted as near complete MAGs, according to MIMAG thresholds [15]. Taxonomy was assigned to the MAGs with GTDB-Tk [16] v2.4.0 using the classify workflow.

Publicly available metagenomes

We downloaded publically available data from metagenomic studies with the highest possible sequencing depth available, spanning eight different habitats, encompassing five non-soil environments: human gut, pig gut, seawater, wastewater, and activated sludge, and four soil environments: agricultural soil, forest soil, grassland soil, and rhizosphere soil (Table S1). We evaluated the metagenomic coverage, diversity, and required sequencing depth as described above.

Statistical analysis

Statistical analysis was done with R (v4.4.1). Differences between groups were assessed using a two-sided Wilcoxon rank-sum test. Linear regressions were fitted using the lm function, and Pearson correlation coefficients were computed with the cor.test function. Logistic regressions were fitted with the nls function. All functions are from the stats R package (v4.4.1).

REFERENCES

1. Krueger, Felix, Frankie James, Phil Ewels, Ebrahim Afyounian, Michael Weinstein, Benjamin Schuster-Boeckler, Gert Hulselmans, and sclamons. 2019. "FelixKrueger/TrimGalore." Zenodo. https://doi.org/10.5281/zenodo.7598955

2. Martin, Marcel. 2011. "Cutadapt removes adapter sequences from high-throughput sequencing reads." *EMBnet.Journal* 17: 10–12. https://doi.org/10.14806/ej.17.1.200

3. Rodriguez-R, Luis M., Santosh Gunturu, James M. Tiedje, James R. Cole, and Konstantinos T. Konstantinidis. 2018. "Nonpareil 3: fast estimation of metagenomic coverage and sequence diversity." *mSystems* 3: e00039-18. https://doi.org/10.1128/msystems.00039-18

4. Li, Dinghua, Chi-Man Liu, Ruibang Luo, Kunihiko Sadakane, and Tak-Wah Lam. 2015. "MEGAHIT: an ultra-fast single-node solution for large and complex metagenomics assembly via succinct de Bruijn graph." *Bioinformatics* 31: 1674–76. https://doi.org/10.1093/bioinformatics/btv033

5. Saheb Kashaf, Sara, Alexandre Almeida, Julia A. Segre, and Robert D. Finn. 2021. "Recovering prokaryotic genomes from host-associated, short-read shotgun metagenomic sequencing data." *Nature Protocols* 16: 2520–2541. https://doi.org/10.1038/s41596-021-00508-2

6. Aroney, Samuel T N, Rhys J P Newell, Jakob N Nissen, Antonio Pedro Camargo, Gene W Tyson, and Ben J Woodcroft. 2025. "CoverM: read alignment statistics for metagenomics." *Bioinformatics* 41: btaf147. https://doi.org/10.1093/bioinformatics/btaf147

7. Hyatt, Doug, Gwo-Liang Chen, Philip F. LoCascio, Miriam L. Land, Frank W. Larimer, and Loren J. Hauser. 2010. "Prodigal: prokaryotic gene recognition and translation initiation site identification." *BMC Bioinformatics* 11: 119. https://doi.org/10.1186/1471-2105-11-119

8. Steinegger, Martin, and Johannes Söding. 2018. "Clustering huge protein sequence sets in linear time." *Nature Communications* 9: 2542. https://doi.org/10.1038/s41467-018-04964-5

9. Langmead, Ben, and Steven L. Salzberg. 2012. "Fast gapped-read alignment with Bowtie 2." *Nature Methods* 9: 357–359. https://doi.org/10.1038/nmeth.1923

10. Li, Heng, Bob Handsaker, Alec Wysoker, Tim Fennell, Jue Ruan, Nils Homer, Gabor Marth, Goncalo Abecasis, Richard Durbin, and 1000 Genome Project Data Processing Subgroup. 2009. "The sequence alignment/map format and SAMtools." *Bioinformatics* 25: 2078–2079. https://doi.org/10.1093/bioinformatics/btp352

11. Nissen, Jakob Nybo, Joachim Johansen, Rosa Lundbye Allesøe, Casper Kaae Sønderby, Jose Juan Almagro Armenteros, Christopher Heje Grønbech, Lars Juhl Jensen, *et al.* 2021. "Improved metagenome binning and assembly using deep variational autoencoders." *Nature Biotechnology* 39: 555–560. https://doi.org/10.1038/s41587-020-00777-4

12. Chklovski, Alex, Donovan H. Parks, Ben J. Woodcroft, and Gene W. Tyson. 2023. "CheckM2: a rapid, scalable and accurate tool for assessing microbial genome quality using machine learning." *Nature Methods* 20: 1203–1212. https://doi.org/10.1038/s41592-023-01940-w

13. Olm, Matthew R, Christopher T Brown, Brandon Brooks, and Jillian F Banfield. 2017. "dRep: a

tool for fast and accurate genomic comparisons that enables improved genome recovery from metagenomes through de-replication." *The ISME Journal* 11: 2864–2868. https://doi.org/10.1038/ismej.2017.126

14. Jain, Chirag, Luis M. Rodriguez-R, Adam M. Phillippy, Konstantinos T. Konstantinidis, and Srinivas Aluru. 2018. "High throughput ANI analysis of 90K prokaryotic genomes reveals clear species boundaries." *Nature Communications* 9: 5114. https://doi.org/10.1038/s41467-018-07641-9

15. Bowers, Robert M., Nikos C. Kyrpides, Ramunas Stepanauskas, Miranda Harmon-Smith, Devin Doud, T. B. K. Reddy, Frederik Schulz, *et al.* 2017. "Minimum information about a single amplified genome (MISAG) and a metagenome-assembled genome (MIMAG) of bacteria and archaea." *Nature Biotechnology* 35: 725–731. https://doi.org/10.1038/nbt.3893

16. Chaumeil, Pierre-Alain, Aaron J Mussig, Philip Hugenholtz, and Donovan H Parks. 2022. "GTDB-Tk v2: memory friendly classification with the genome taxonomy database." *Bioinformatics* 38: 5315–5316. https://doi.org/10.1093/bioinformatics/btac672

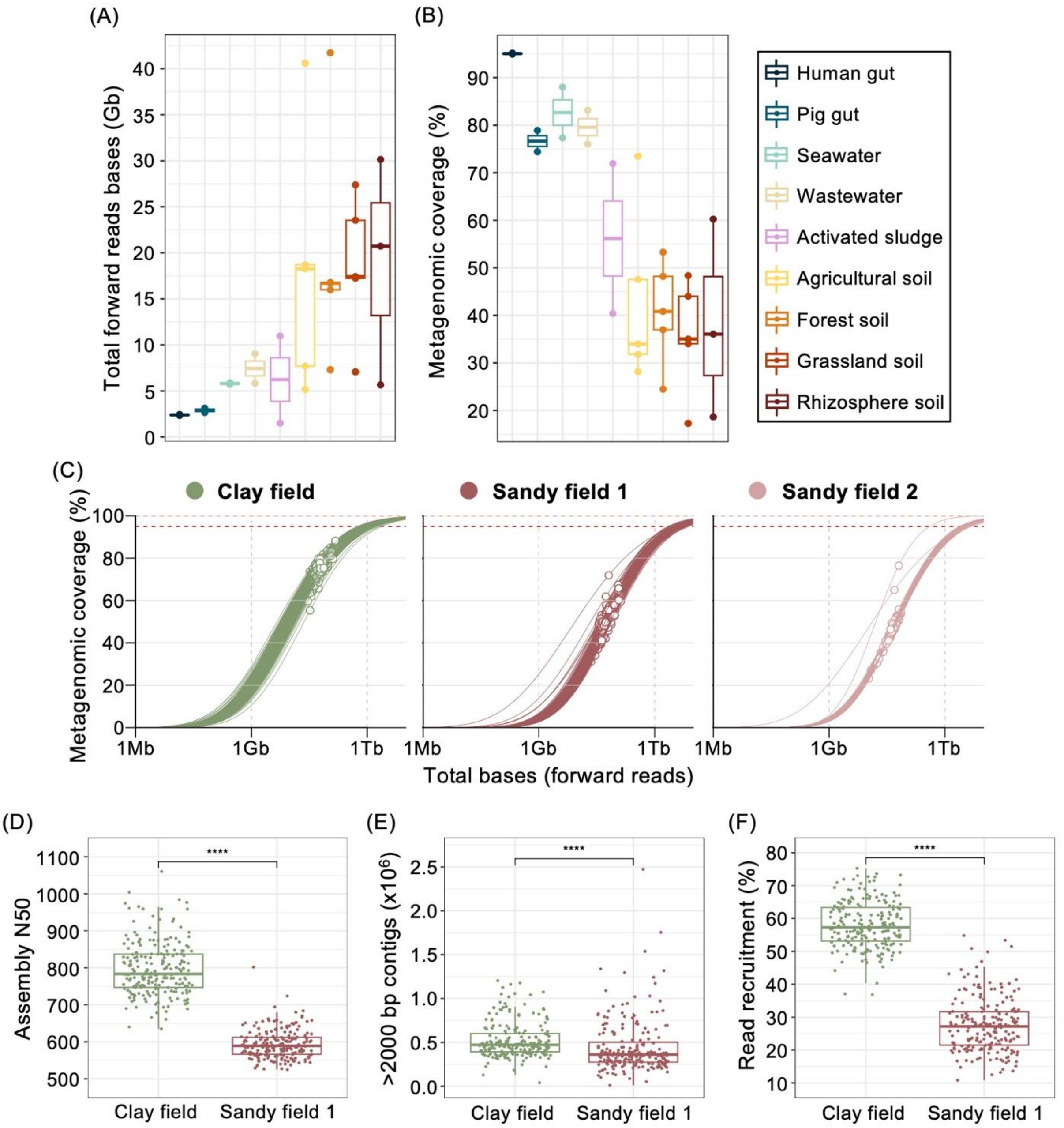

Figure S1 Metagenomic sequencing depth and coverage varies in different environments and greatly impacts assembly quality. (A) Sequencing depth as measured by the total number of gigabases (Gb) in the clean forward reads and (B) Nonpareil-estimated metagenomic coverage of public metagenomic samples. (C) Nonpareil curves for each sample, showing the estimated metagenomic coverage as a function of the sequencing depth. Points represent the actual sequencing depth and estimated coverage of each sample, while lines represent projected and rarefied sequencing depths. (D–F) Samples from clay field and sandy field 1 was subjected to *de-novo* assembly. (D) Assembly N50, (E) number of contigs longer than 2000 bp, and (F) percentage of reads mapping to > 2000 bp assembly contigs. For A,B,D–F, horizontal lines indicate the

median; box boundaries indicate the interquartile range; whiskers represent values within 1.5× the interquartile range of the first and third quartiles. Statistics based on two-sided Wilcoxon rank-sum test. **** $p \leq 0.0001$.

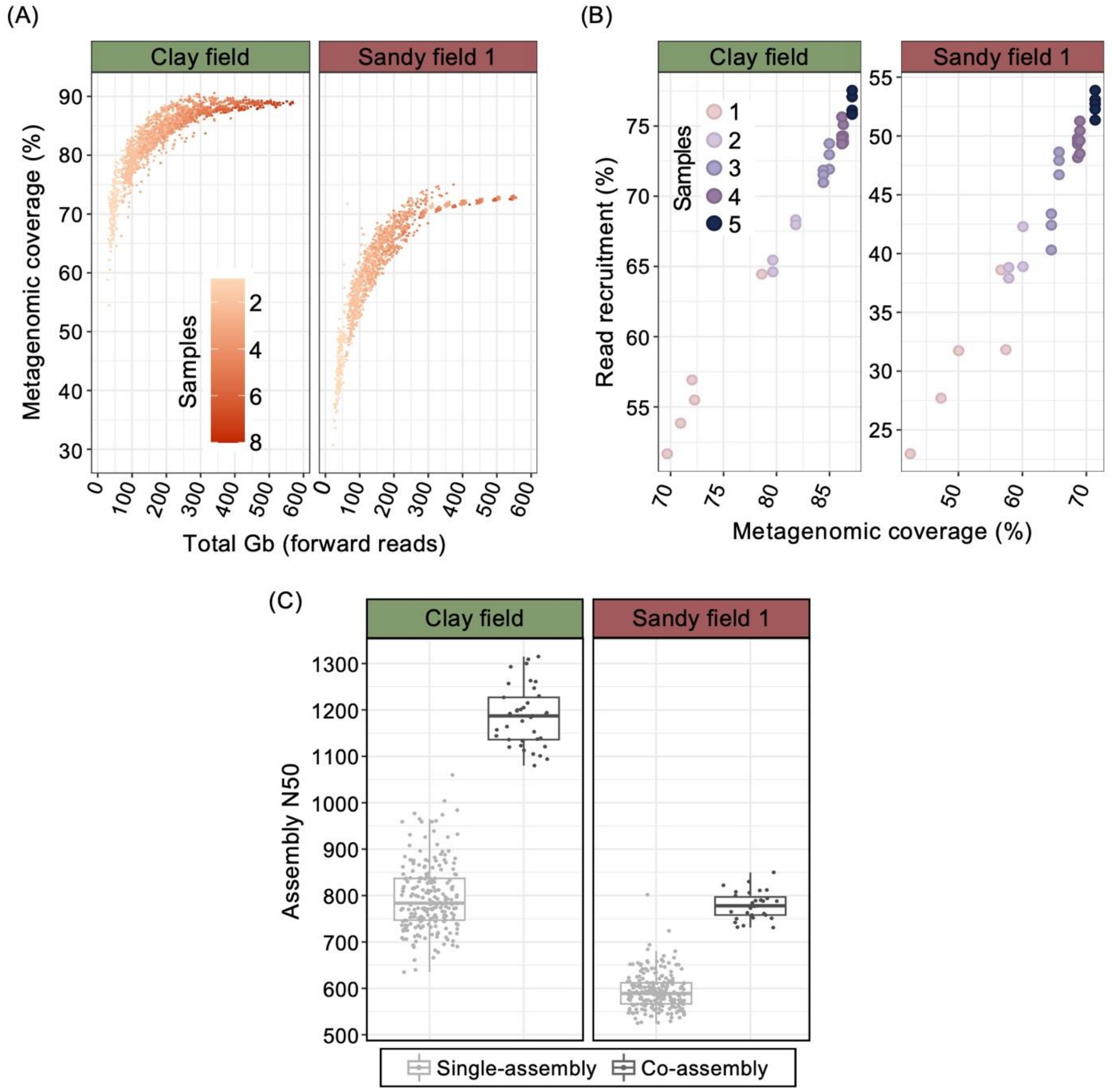

Figure S2 Co-assembly increases metagenomic coverage and read recruitment to assembly. (A) Reads from 2–8 samples were *in silico* combined to evaluate the effect on metagenomic coverage in comparison to single samples. Points show the total size of the combined forward reads in gigabases (Gb), and the resulting metagenomic coverage depending on how many samples are combined. (B) Assemblies were carried out for five samples for each field. Samples were single-assembled and assembled in 2-, 3-, 4-, and 5-sample co-assemblies. Points represent the percentage of reads mapping to > 2000 bp assembly contigs, which increases with metagenomic coverage, and the number of samples co-assembled. (C) N50 for 5-sample co-assemblies and single-assemblies of 340 samples. Horizontal lines indicate the median; box boundaries indicate the interquartile range; whiskers represent values within 1.5× the interquartile range of the first and third quartiles.

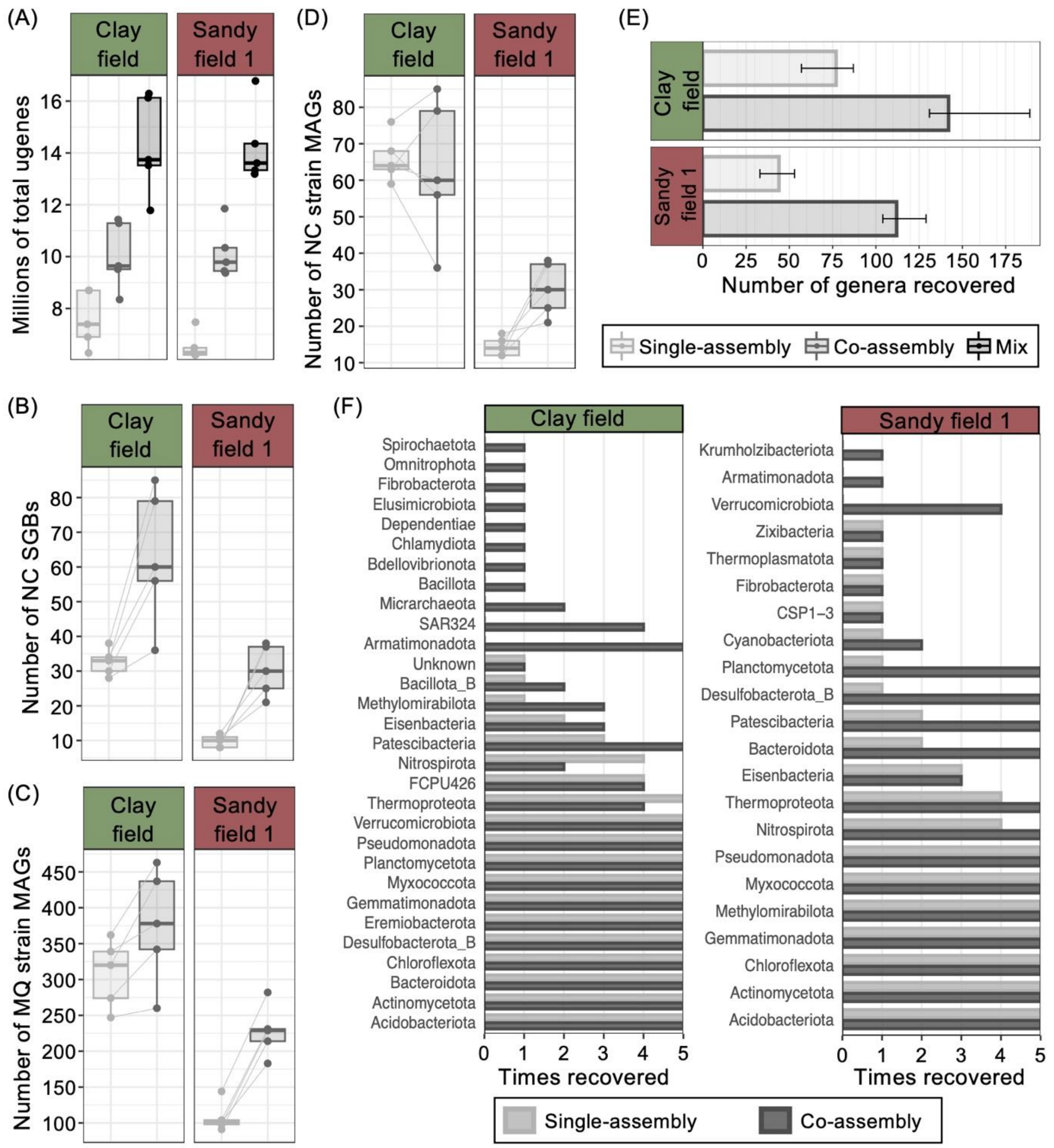

Figure S3 Co-assembly improves recovery of microbial genomes and genes. Metagenome-assembled genomes (MAGs) were recovered from five 5-sample co-assemblies, and the single-assemblies for the in total 25 samples participating in each of the five co-assemblies. The number of (A) unique genes in millions, (B) near complete (NC) species-level MAGs, (C) medium quality (MQ) and (D) NC strain-level MAGs, and (E) genera recovered from single-assemblies and co-assemblies, respectively. (F) The number of times each phylum was recovered, comparing single-sample assemblies *vs.* co-assemblies of the same samples. For A–D, horizontal lines indicate the median; box boundaries indicate the interquartile range; whiskers represent values within 1.5× the interquartile range of the first and third quartiles. Lines connect points representing

the same group of samples single-assembled and co-assembled. For E, bars represent the median number of genera recovered, while whiskers represent the minimum and the maximum values.
